# Active Aging in Very Old Age and the Relevance of Psychological Aspects

**DOI:** 10.3389/fmed.2017.00181

**Published:** 2017-10-30

**Authors:** Constança Paúl, Laetitia Teixeira, Oscar Ribeiro

**Affiliations:** ^1^Center for Health Technology and Services Research, Institute of Biomedical Sciences Abel Salazar (CINTESIS-ICBAS), University of Porto, Porto, Portugal; ^2^Department of Education and Psychology, University of Aveiro, Aveiro, Portugal; ^3^Higher Institute of Social Service of Porto (ISSSP), Senhora da Hora, Portugal

**Keywords:** active aging, World Health Organization, confirmatory factor analysis, health, psychological determinants

## Abstract

**Background:**

Active aging encompasses a socially and individually designed mix of different domains that range from personal and familial, to social and professional. In being a key policy concept often focused on the young-old individuals, efforts in studying its dimensions in advanced ages have seldom been made. Nevertheless, there is a recognized need to promote adequate responses to the growing number of individuals reaching advanced ages and to recognize their specific dependability on health-related aspects, services attendance, social interactions, or on psychological characteristics for what it means to “age actively.”

**Objective and methods:**

This study provides a secondary analysis of data and follows the preceding work on the operationalization of the World Health Organization’s (WHO) active aging model by means of an assessment protocol to measure which variables, within the model’s determinants, contribute the most for an active aging process (1). Authors used the achieved model (composed by six factors: health, psychological component, cognitive performance, social relationships, biological component, and personality) and performed multi-group analysis of structural invariance to examine hypothetical differences between age groups (<75 years vs. ≥75 years) and to contrast obtained findings with the originally achieved model for the total sample (1,322 individuals aged 55 +).

**Results:**

The structural covariances for the two age groups were statistically different. The comparison of components between age groups revealed a major relevance of the psychological component for the older age group.

**Conclusion:**

These findings reinforce the importance of psychological functioning in active aging in oldest old, and the need for further research on specific psychological features underlying the subjective meaning of active aging in more advanced ages.

## Introduction

The concept of Active Aging is defined as “…*the process of optimizing opportunities for health, participation and security in order to enhance quality of life as people age*” (2). In being of incontestable importance as a fundamental policy concept in Europe, efforts to increase its empirical evidence in terms of operative definition and criteria have received growing attention worldwide over the last years [e.g., Ref. (3–5)].

The active aging model as presented by the World Health Organization (WHO) (2) encompasses six groups of determinants, each one including several features: (1) availability and use of health and social services (e.g., health promotion and prevention; continuous care); (2) behavioral determinants (e.g., exercise and physical activity; drinking and smoking habits; feeding; medication); (3) personal determinants (biology and genetics, and psychological characteristics); (4) physical environment (e.g., safety houses, low pollution levels); (5) social determinants (e.g., education, social care), and (6) economic determinants (e.g., wage, social security). This group is complemented by two crosscutting determinants—gender and culture. According to this model, the key elements of active aging are (1) autonomy, which is the perceived ability to control, cope with, and make decisions about how one lives on a day-to-day basis, according to personal rules and preferences; (2) independence, which refers to the ability one has to perform functions related to daily living, i.e., the capability of living in the community with no and/or little help from others; (3) quality of life; and (4) healthy life expectancy, which refers to how long people can expect to live in the absence of disabilities. The main pillars of the model are participation, health, and security. More recently, a fourth pillar has been added to the model: lifelong learning (6).

Presently, active aging appears mostly as an outcome of several determinants that are expected to help professionals and researchers to recognize particular profiles that are more at risk or, on the contrary, are more favorable to age actively. Since the majority of definitions of active aging are narrow and primarily concerned with the young-old [cf. (7)], the need for further investigating the concept’s operationalization in the old-old has been increasingly recognized (8), for instance, building on and expanding the classic WHO’s definition of active aging, proposed a set of principles as the basis for a wide-ranging strategy on active aging that incorporate, among others, the need for encompassing all older people, including those who are frail and dependent, i.e., those who are more likely to be older and experiencing sizeable losses in cognitive and physical potential. On this specific matter (9), in an Age UK report entitled *Improving later life: Understanding the oldest old* had already stressed the importance of fully integrating the older population into an active aging strategy that includes both prevention at earlier stages of the life course as well as into old age, and fast remedial action when autonomy is threatened.

By enlighten the active aging model in the oldest old (75+), this paper aims to test the statistical significance of observed differences in the structural weights of health, psychological component, cognitive performance, social relationships, biological component, and personality factors, across age groups.

## Materials and Methods

### Data Collection

This study was conducted in several Portuguese regions, including mainland territory and the Azores and Madeira islands, and is part of a large Portuguese project on active aging entitled “DIA: From Incapacity to Activity—The Challenge of Ageing” that included a cross-sectional survey of community-dwelling individuals aged 55 years old and over. Participants were recruited randomly through announcements in local media, local agencies (e.g., seniors clubs) and NGO’s, and also recurring to the snowball method through which effective participants indicated other potential subjects who were in similar conditions. Trained researchers conducted the interviews and followed a structured questionnaire that included question on demographic, psychological, and social aspects. All interviews took place in local community facilities (e.g., parish hall) or at the participants’ homes.

### Measures

Different groups of determinants of active aging were assessed by means of an extensive protocol that was developed considering literature review of most common tests used in Gerontology and Geriatrics, and following previous experience with the European Survey of Aging Protocol [ESAP (10)]. Along with socio demographic characteristics (gender, age, education, and income), information was obtained on cognitive functioning by means of the Mini-Mental State Examination [MMSE (11, 12)], social network (family, friends, confidents) was assessed with the Lubben Social Network Scale [LSNS (13)], psychological distress was measured with the General Health Questionnaire [GHQ-12 (14)], optimism was assessed with the Life Orientation Test-Revised [LOT-R (15, 16)], personality (neuroticism, extraversion, openness to experience) was evaluated with the NEO Personality Inventory (17), happiness was assessed with a single question retrieved from QBE/F (18) and environment domain of quality of life measured with World Health Organization Quality of Life-BREF [WHOQOL-BREF (19, 20)]. Bio-behavioral measures included pulmonary function and grip strength, which were calculated using a standard “Mini Peak-Flow Meter” (Datosprir Peak-10, Sibelmed) and an electronic dynamometer (Grip-D, TAKEI Scientific Instruments Co., Ltd.), respectively. Finally, health status and physical condition were assessed by self-report indicators of health condition (determined by a standard health-rating item: “In general, how would you rate your health?”), illness (sum of self-reported health problems), sleep problems, subjective physical activity (determined by the item: “In general, how would you rate your physical condition?”), ADL, and loneliness. A detailed description of the assessment protocol (*Protocol of Assessment of Active Aging*—*P3A*) comprising all instruments used for each of the WHO’s active aging model determinants can be found elsewhere (1, 21). Table 1 presents the variables used and its correspondent coding.

**Table 1:** Definition of variables.

Variable	Coding
Subjective health	1 = Very good; 2 = Good; 3 = Reasonable; 4 = Poor; 5 = Very poor
Sleep problems	0 = No; 1 = Yes
Subjective physical activity	1 = Very good; 2 = Good; 3 = Reasonable; 4 = Poor; 5 = Very poor
ADL	0 = With difficulties; 1 = Without difficulties
Illness	0 = None; 1 = 1 illness; 2 = 2 illness; 3 = illness; 4 = 4 or more illness
Psychological distress^a^	1 = <9; 2 = [9,12]; 3 = [12,16]; 4 = ≥16
Happiness	1 = Nothing; 2 = 2; 3 = 3; 4 = Very
Optimism^a^	1 = <11; 2 = [11,13]; 3 = [13,15]; 4 = ≥15
Quality of life^a^	1 = <24; 2 = [24,26]; 3 = [26,29]; 4 = ≥29
Loneliness	0 = Yes; 1 = No
Cognitive impairment^a^	1 = <25; 2 = [25,28]; 3 = [28,30]; 4 = ≥30
Income	1 = ≤386€; 2 = 386–772€; 3 = 772–1158€; 4 = > 1,158€
Education level	1 = No formal; 2 = Primary; 3 = 5–8 years; 4 = 9–12 years; 5 = University
Peak Flow^a^	1 = <180; 2 = [180,250]; 3 = [250,340]; 4 = ≥340
Grip Strength^a^	1 = <18.3; 2 = [18.3,22.9]; 3 = [22.9,29.0]; 4 = ≥29.0
Family^a^	1 = <9; 2 = [9,11]; 3 = [11,13]; 4 = ≥13
Friends^a^	1 = <5; 2 = [5,8]; 3 = [8,10]; 4 = ≥10
Confidents^a^	1 = <4; 2 = [4,7]; 3 = [7,9]; 4 = ≥9
Neuroticism^a^	1 = <30; 2 = [30,34]; 3 = [34,37]; 4 = ≥37
Extraversion^a^	1 = <39; 2 = [39,41]; 3 = [41,44]; 4 = ≥44
Openness to Experience^a^	1 = <35; 2 = [35,37]; 3 = [37,40]; 4 = ≥40

*^a^Quartiles*.

### Ethical Procedure

The study was submitted to the ethical commission of UNIFAI/ICBAS-UPORTO. All the participants signed the informed consent form that was developed according the Declaration of Helsinki.

### Statistical Analysis

#### Invariance Analysis Methods

Confirmatory factor analysis (CFA) models of factorial invariance enable one to test explicitly the structure of a model or its individual parameters for equivalence across subgroups or conditions (22). Two primary models were tested: Model 1 was the baseline model with all parameters allowed to vary across groups and resulted in the first chi-square value for comparison with Model 2, which imposed the equality of factor loadings constraint across groups. The difference in the Model 2 and Model 1 chi-squares was used to evaluate overall invariance. Groups were defined regard to age: group 1: <75 years and group 2: ≥75 years. To ensure that statistically significant results were not due to model misfit, a variety of fit indexes were examined, including the chi-square goodness-of-fit test, comparative fit index (CFI), and goodness-of-fit index (GFI).

#### Exploratory and CFA

In the case of a significant result in the invariance analysis, the factor structure of P3A for the age group 75+ was examined by exploratory factor analysis (EFA) and CFA, using the same methodology as described in the previous work [cf. (1)]. In the EFA, a principal-components extraction with Varimax rotation was used. In the CFA, satisfaction scores for each dimension were obtained using factor score regressions. A nested models approach to test alternatives to the full model was used to obtain the final model. Goodness-of-fit criterion was used to evaluate the model fit.

A significant level of 0.05 was used in all analysis, conducted using SPSS 20 and AMOS 19 for Windows.

## Results

### Sample Characteristics

The sample comprised 1,322 persons with an average age of 70.4 years (SD 8.7 years; age range 55–101 years old). Most participants were women (71.1%; *n* = 939). More than half of the participants were married/partnered (55.7%; *n* = 729), 30.6% (*n* = 400) were widowed, 8.7% (*n* = 114) were single, and 5.0% (*n* = 65) were divorced. Almost a quarter of the sample (24.7%) lived alone. As for the educational level, 55.3% attended primary school (4 years schooling), 19.1% never attended school, 17.8% had completed high school, and only a minority presented a trade qualification or university degree (7.7%). Almost half of the participants (49.6%) earned per month the equivalent or less than the minimum national wage (around 400 Euros). Table 2 presents the sample characteristics (total and subgroups).

**Table 2:** Sample characteristics (total and subgroups).

	Total (*N* = 1,321)	≥75 years (*N* = 427)
	*n* (%)	*n* (%)
**Gender**		
Male	382 (28.9)	129 (30.3)
Female	939 (71.1)	297 (69.7)
**Age**		
Mean (SD)	70.4 (8.7)	80.3 (4.6)
**Marital status**		
Married	729 (55.7)	152 (36.1)
Widow(er)	400 (30.6)	206 (48.9)
Single	114 (8.7)	44 (10.5)
Divorced	65 (4.9)	19 (4.5)
**Education**		
Illiterate	249 (19.1)	122 (29.3)
4 years education	722 (55.3)	239 (57.3)
5–8 years education	80 (6.1)	27 (6.5)
9–12 years education	153 (11.7)	17 (4.1)
High education	101 (7.7)	12 (2.9)

### Multigroup Analysis of Structural Invariance

To test the invariance across age groups, we conducted a multigroup analysis of structural invariance. The Model 1 (baseline model—equal pattern) had CFI and GFI of 0.890 and 0.921, respectively. The respective baseline chi-square was 825.022 with 348 df (Table 3). In the Model 2, each item-factor loading was forced to be equal across the two age groups. Model 2 had CFI and GFI of 0.878 and 0.916, respectively. The chi-square for Model 2 is 891.526 with 363 df (Table 3). The Model 2 was nested within Model 1 [the set of parameters estimated in the more restrictive model (Model 2) was a subset of the parameters estimated in the less restrictive model (Model 1)]. Thus, the chi-square difference between Model 2 and Model 1 provided a test of the pre-condition for testing the invariance of structural weights. The model appeared not to be invariant across subgroups (*p* < 0.001), suggesting different structure for different age groups. All following analyses were performed only for people with 75 or more years (*N* = 269).

**Table 3:** Structural invariance analysis.

No.	Model	χ^2^	df	*p*	CFI	GFI	_Δ_χ^2^	Δdf	*p*
1	Unconstrained	825.022	348	<0.001	0.890	0.921			
2	Measurement weights	891.526	363	<0.001	0.878	0.916	66.504	15	<0.001

*χ^2^, Chi-square test; df, degree of freedom; p, p-value; CFI, comparative of fit index; GFI, goodness-of-fit index*.

### Exploratory Factor Analysis

The factor structure was examined by principal-components extraction with Varimax rotation for the sub-sample with 75+ years (*n* = 269). The Bartlett Sphericity test (*p* < 0.001) and the Kaiser–Meyer–Olkin (KMO = 0.798) test indicate that factor analysis seemed to be highly adjusted to this analysis. Six distinct factors, presented in Table 4, were revealed explaining 55.5% of total variance. The final structure was Factor 1 (psychological component): six variables load heavily of this factor (psychological distress, happiness, optimism, neuroticism, quality of life—environment, and loneliness), which account for 12.9% of the total variance; Factor 2 (health component): this factor comprises four variables (subjective health, subjective physical condition, ADL, and illness) and explained 12.0% of total variance; Factor 3 (cognitive performance): three questions have their highest loadings on this factor (cognitive impairment, income, and education level) and explained 9.6% of total variance; Factor 4 (biological component): this factor comprises only two variables (peak flow and grip strength) and explained 7.4% of total variance; Factor 5 (social relationship component): three variables have their highest loadings on this factor (family, friends, and confidence), accounting for 6.9% of total variance; and Factor 6 (personality component): the last factor contains only two variables (extraversion and openness to experience) and explained 6.6% of total variance.

**Table 4:** Factor Structure of P3A for people aged 75 or more years: exploratory factor analysis.

Questions	Component					
	1	2	3	4	5	6
Psychological distress	***−0.540***	0.413	−0.221	−0.072	−0.116	0.166
Happiness	***0.614***	−0.259	0.111	−0.189	0.265	−0.084
Optimism	***0.708***	−0.069	−0.011	0.037	−0.016	0.095
Neuroticism	***−0.664***	0.015	−0.157	−0.225	0.143	−0.017
Quality of life	***0*.*649***	−0.145	0.219	0.044	−0.002	0.121
Loneliness	***0*.*519***	−0.032	0.048	0.180	0.249	−0.303
Subjective health	−0.368	***0*.*702***	−0.163	−0.056	−0.063	0.042
Subjective physical condition	−0.255	***0*.*761***	−0.071	−0.089	−0.031	−0.055
ADL	−0.034	***−0*.*720***	0.215	−0.036	0.026	−0.048
Illness	0.064	***0*.*446***	0.222	−0.270	0.065	0.353
Cognitive impairment	0.255	0.030	***0*.*691***	0.150	0.102	−0.111
Income	0.056	−0.143	***0*.*711***	0.104	0.097	−0.028
Education level	0.137	−0.151	***0*.*804***	0.052	−0.097	0.009
Peak flow	0.134	0.006	0.303	***0*.*657***	−0.007	0.010
Handgrip	0.102	−0.128	0.064	***0*.*799***	0.103	0.005
Social relationship—family	0.025	−0.068	0.105	−0.161	***0*.*782***	−0.101
Social relationship—friends	0.070	−0.347	−0.059	0.245	***0*.*294***	0.139
Social relationship—confidents	0.044	−0.018	−0.002	0.257	***0*.*687***	0.099
Extraversion	0.266	−0.393	−0.111	−0.251	0.063	***0*.*414***
Openness to experience	0.005	−0.084	−0.077	0.040	0.107	***0*.*725***
Sleep problems	−0.100	0.359	−0.013	0.118	−0.220	***0.593***
% of variance explained	12.9	12.0	9.6	7.4	6.9	6.6

*Bold-italic indicates to show in which factor each item present higher factor loading, without signficant level associated*.

When comparing to the six-factor structure model for P3A originally obtained for the pooled sample (*n* = 925) which explained 54.6% of the total variance [cf. (1)], the achieved model for this age group reveals that the “psychological component” is the main factor associated with active aging, followed by “health component,” and that these previously occupied reverse positions (health component was in first place and explained 11.6% of total variance, followed second by the psychological component which explained 11.2% of total variance). All the other factors in the original model maintained a similar order to the current one (cognitive performance explained 10.6% of total variance; biological component explained 7.7% of total variance; social relationships explained 6.6% of total variance; and personality explained 6.6% of total variance).

### Confirmatory Factor Analysis

**Figure 1:**
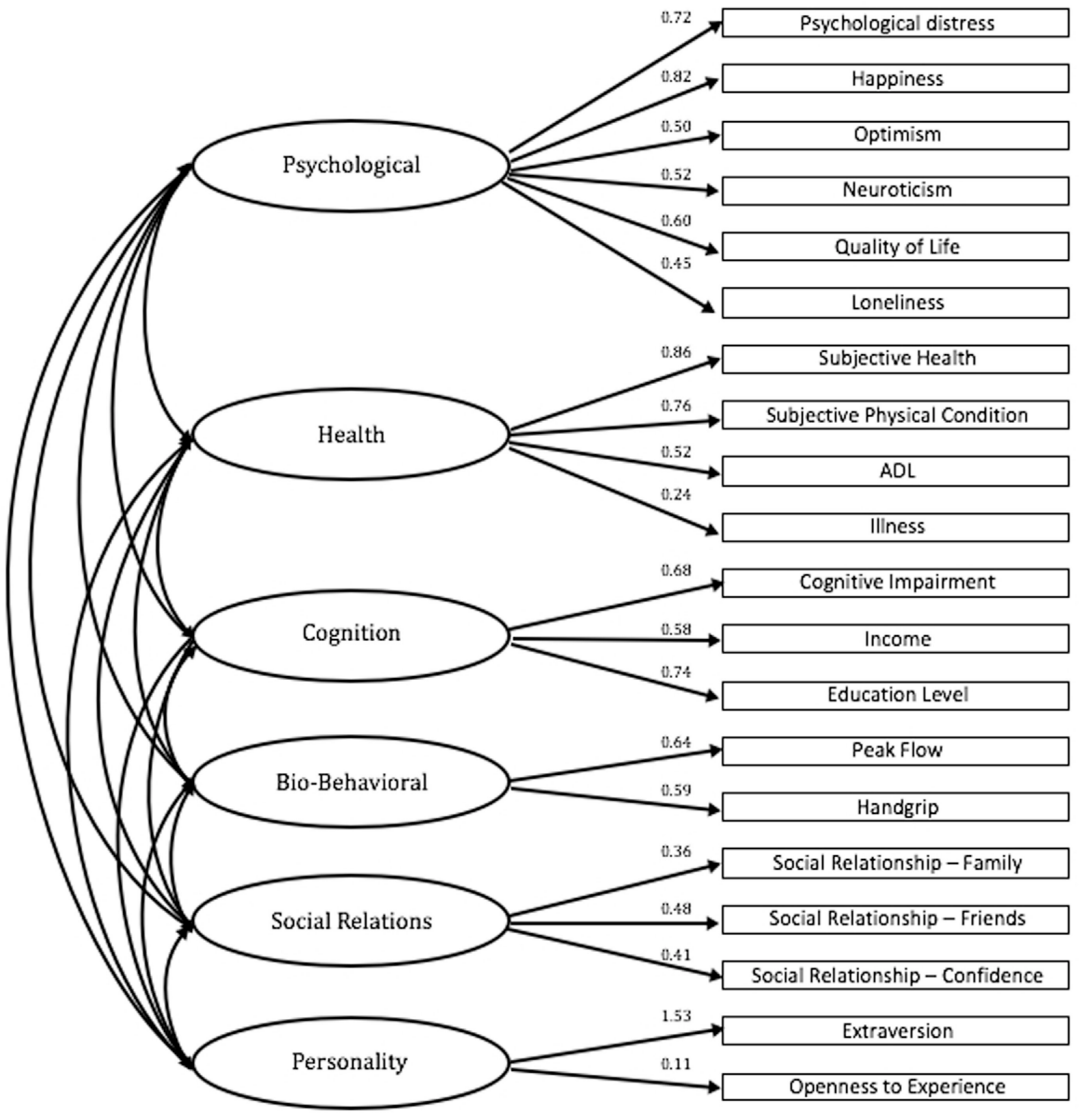
Factor structure model of P3A for those aged 75+ years old: confirmatory factor analysis.

We analyzed the full six-factor model for the 21 variables derived from the EFA (Table 4). For the final model, we used a nested models approach to test alternatives to the full model, eliminating of item “sleep problems.” The Confirmatory Factor analyses structure describes adequately the six factors reinforcing the adequacy of the proposed model. The goodness-of-fit indices suggest that the structure can be adequately described by the six correlated factors that are graphically presented in Figure 1 [χ^2^ (155) = 226.700, *p* < 0.001; CFI = 0.928 and GFI = 0.924; covariance and error estimates were omitted of the figure]. Although the personality component was not significant, it was decided to keep the same model in order to preserve the original structure of the model.

## Discussion

In overall, this study’s main finding points to the prominence of the psychological component in defining active aging in the oldest age group as it adds evidence to the particular value of psychological functioning (namely absence of psychological distress, presence of happiness and optimism, low neuroticism, good quality of life, and low loneliness) in allowing an active involvement with life. The comparison of components between age groups revealed its major relevance on the achieved model for the 75+ group as it now explains 12.9% of total variance, followed by the health component that explains 12% of total variance. In the originally achieved model for the total sample [cf. (1)], the health component occupied first place in the factor structure model for P3A and explained 11.6% of total variance, whereas the psychological component, occupying second place, explained 11.2% of the total variance.

This observed change in the relative load of these two factors reinforces the notion that rather than health problems that most older people face, the differentiating aspect between those individuals who are aging actively from those who do not may be rooted in psychological characteristics and strengths. Traditionally, the majority of definitions of “successful aging” are based on the absence of disability with lesser inclusion of psychological variables (23). However, it is not surprising the relevance of psychological variables later in life and in predicting quality of life in older adults by maximizing one’s self-efficacy and resilience (24) as they are involved in emotional regulation and related to health by multiple pathways: at physiological level through immune system and at emotional level through experiences and generation of psychological resources and eliciting social support to deal with adversity, and finally at motivation level through health-relevant behaviors (25). Furthermore “the ability to maintain an awareness of our positive emotions in the face of life inevitable difficulties, including challenges to health, may be a hidden key to resilience as we age.” (26).

There is an aging paradox referring to the stability or increase of affective wellbeing across adulthood while, at the same time, cognition or physical health decline (27). In fact, there is a wide consensus that older people are generally more focused on emotional issues and report more positive emotions than the younger ones [e.g., Ref. (27–29)]. This psychological capacity (e.g., happiness, optimism, quality of life and less distress, neuroticism, or loneliness) may be what makes the difference between those aging actively and those less active, in times when having health-related issues is the norm, more than the exception. Regardless of still being to some extent autonomous while living in the community, old people may gradually lose their adaptation potential. Active aging discourses that pay a strong emphasis on health and independence, or understand the concept in terms of occupation and “youthful activities” should incorporate a more psychological rooted perspective. By acknowledging the important contributions of psychology in the conceptualization of Active Aging (30), the concept can be encapsulated as being “engaged in life” in more advanced ages and potentially incorporate spiritual and philosophical dimensions (31, 32), which are acknowledged to be of particular relevance in very old ages.

The main contributions of the present paper stress the importance of paying particular attention to the oldest group of people (75+) and emphasize the role of psychological variables in the active aging. The “subjective nature” of the concept of active aging has been already acknowledged by several researchers, including Bowling (33) who found that more than a third of respondents in her study rated themselves as aging “very actively,” and almost a half as “fairly actively.” People based their judgment on: having/maintaining physical health and functioning (43%); keeping leisure and social activities (34%) as well as maintaining mental activity (18%), and social relationships (15%). The subjective meaning of the words “active ageing” was: physical activity; autonomy, interest in life, being able to cope with challenges, and stay aware of the world (34). People seem, therefore, to mix physical, mental, and social factors, and the ability of deciding by their own. By questioning the deterministic view of the WHO model, this study puts an emphasis on the need for introducing, a psychosocial perspective, and take into consideration the pro-active attitude that people claim.

In sum, people should stay engaged with society and at the same time adopt a healthy life style to guarantee the best physical condition. However, the key aspect of the active aging model derives from the balance between individual and social responsibility in aging well as both contribute to aging outcomes. Active aging can only be achieved in contexts that are both supportive and “age friendly,” that value individual choices, and that, in overall, assure an easy access to a wide-ranging set of services according to specific perceived needs (35).

## Conclusion

Based on the present data, psychological aspects proved to be of great relevance for active aging, and corroborate previous research that considers mental health balance as an optimist view of life and cognitive capacity [e.g., Ref. (36, 37)]. It is possible to conclude that although being very important, objective and subjective health seem not to be the main aspects of active aging. Health issues are common at advanced ages and psychological status matters to cope with them. Psychological variables may contribute to more positive attitudes toward health and facilitate functionality, balancing objective measures with subjective ones as supported in the literature [e.g., Ref. (1, 24, 33, 34, 38)]. We advocate greater attention to psychological aspects to foster active aging either by intervening in psychological distress or training coping strategies that help people to keep engaged with life despite the presence of health problems. As the ultimate objective of studying and intervening in very old age is to optimize the aging process and quality of life, this association should by explored in future research as (5) suggested by focusing on the individual and examining the contributions of active aging to life satisfaction and the possible predictive value of coping styles to active aging.

## Ethics Statement

The study was submitted to the ethical commission of UNIFAI/ICBAS-UPORTO. All the participants signed the informed consent form that was developed according the Declaration of Helsinki.

## Author Contributions

CP was responsible for the study conception and design; CP and OR supervised data collection; LT performed the data analysis; CP, LT, and OR wrote the manuscript.

## Conflict of Interest Statement

The authors declare that the research was conducted in the absence of any commercial or financial relationships that could be construed as a potential conflict of interest.
